# Supplementary material for: Low levels of tetracyclines select for a mutation that prevents the evolution of high-level resistance to tigecycline
Source: PLoS Biol. 2022 Sep 28;20(9):e3001808. doi: 10.1371/journal.pbio.3001808 (PMC9550176; doi:10.1371/journal.pbio.3001808)
Supplement: S1 Fig — (PDF) [file pbio.3001808.s013.pdf]

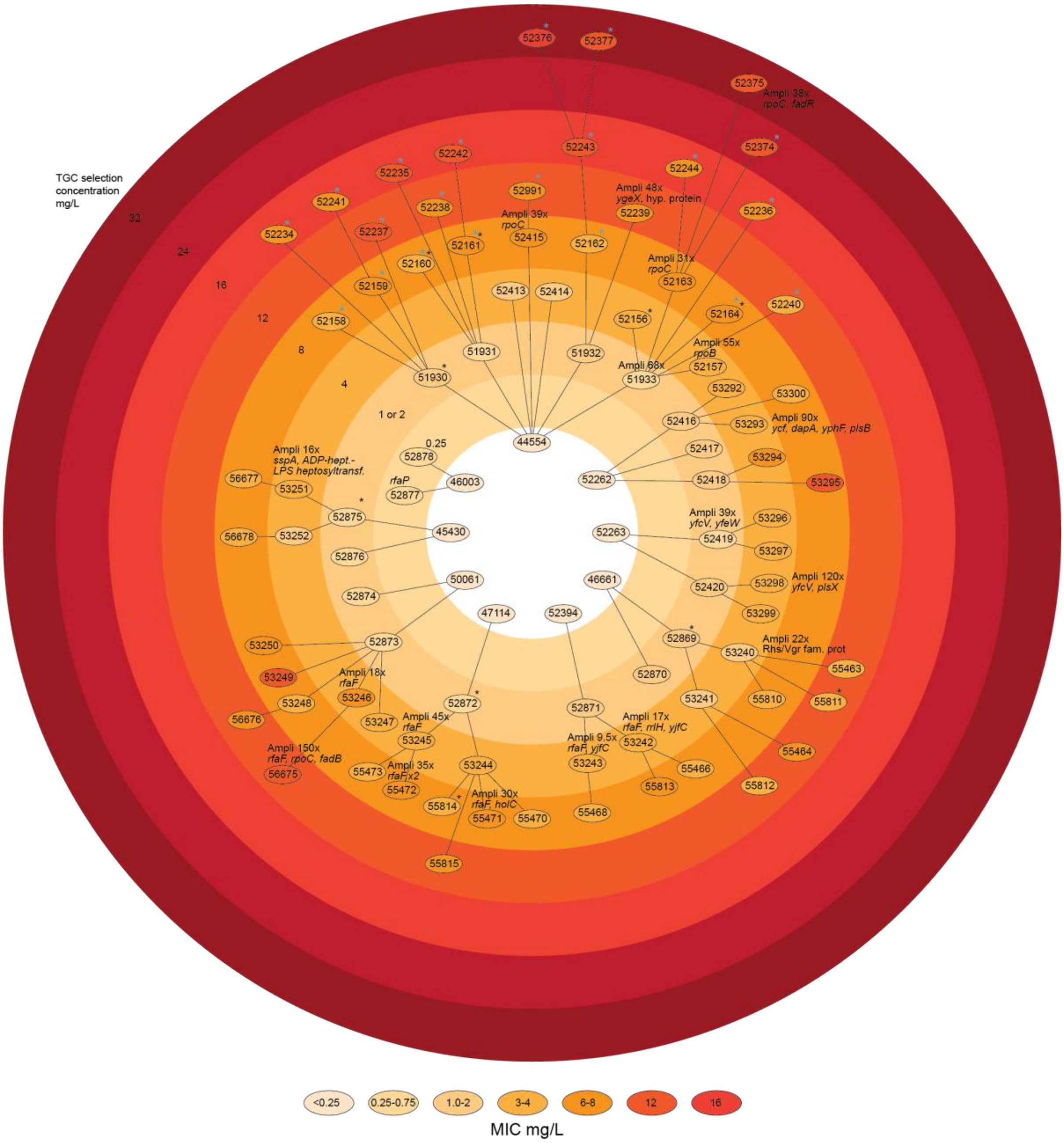

S1 Fig. Step-wise selection of TGC mutants on progressively increasing TGC concentrations. Numbers correlate to DA-collection number. Isolates DA52262 and DA52263 are MG1655 carrying a plasmid with *tet*(A)^{wt} from DA44554. DA52394 and DA50061 are MG1655 carrying plasmids with *tet*(A)^{wt} from DA46661 and DA47114, respectively. Background color represents the concentration of TGC mutants were selected on. Colour of the ellipse represents the MIC of the isolate or mutant. Text above or beside the mutant corresponds to the mutations present in the mutants as well as amplification and *tet*(A)^{wt} copy number per chromosome as determined by whole genome sequencing. For some mutants, amplification of *tet*(A)^{wt} was observed by qPCR (black asterisk). *rpoC* mutations (blue asterisk) were common in mutants deriving from DA44554 and were therefore analysed by local sequencing. ADP-hept.-LPS hept.transf.: ADP-heptose-LPS heptosyltransferase.
