# Supplementary material for: Low levels of tetracyclines select for a mutation that prevents the evolution of high-level resistance to tigecycline
Source: PLoS Biol. 2022 Sep 28;20(9):e3001808. doi: 10.1371/journal.pbio.3001808 (PMC9550176; doi:10.1371/journal.pbio.3001808)
Supplement: S2 Fig — (PDF) [file pbio.3001808.s014.pdf]

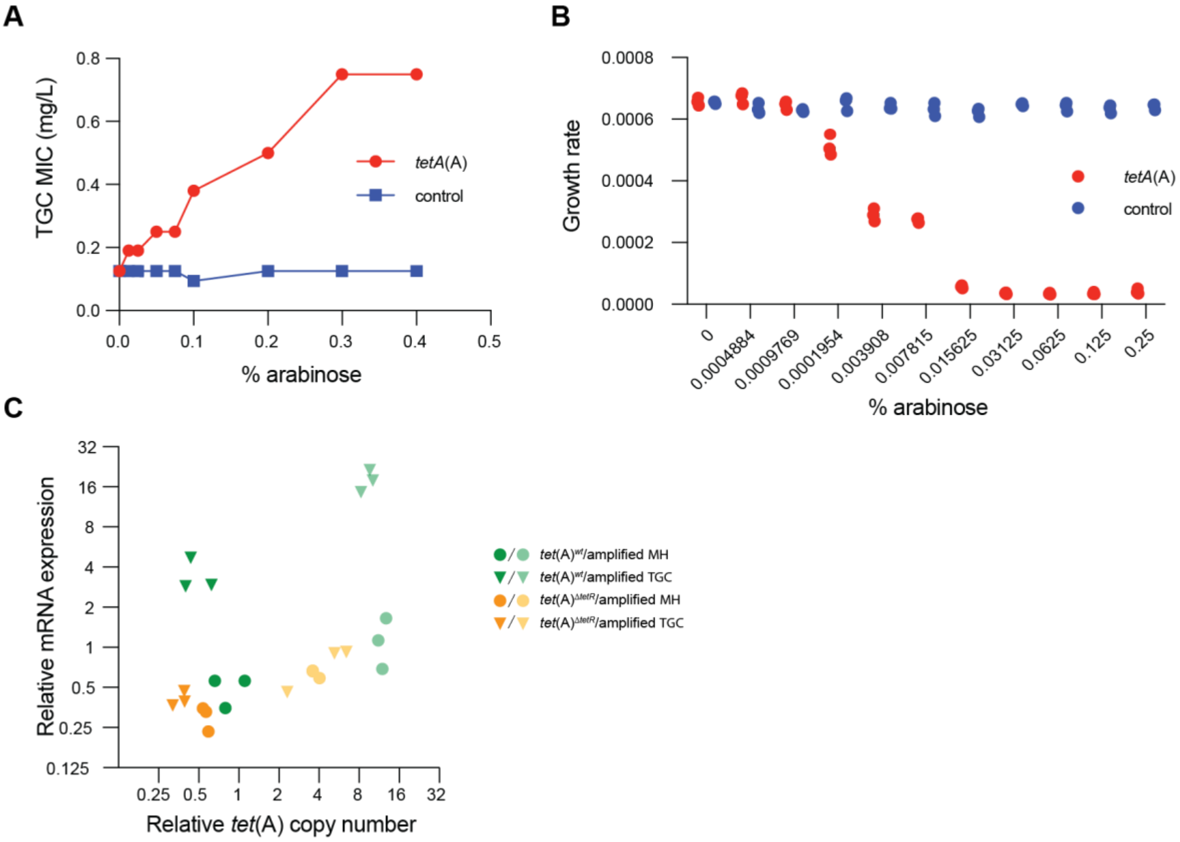

S2 Fig. Effect of *tet*(A) overexpression and copy number increase. A. Effect on TGC MIC of overexpression of *tetA*(A) cloned under an arabinose-inducible promoter (red), compared to an empty vector (blue). Median of three biological replicates. **B.** Effect on fitness (measured as growth rate) of overexpression of *tetA*(A) cloned under an arabinose-inducible promoter (red), compared to an empty vector (blue). Each data point represents a biological replicate, three per condition. **C.** Effect of gene copy number on *tetA*(A) expression. Replicates (3) for *tet*(A)^{wt} and *tet*(A)$^{\Delta tetR}$ are presented in green and red/yellow respectively. For *tet*(A)^{wt}, parental strain without *tet*(A) amplification is DA44554, and mutant with *tet*(A) amplified is DA51930, while for *tet*(A)$^{\Delta tetR}$, parental strain without *tet*(A) amplification is DA33135, and mutant with *tet*(A) amplified is DA35498. Strains were either grown in absence of TGC (MH) or with TGC (1/10th MIC). mRNA expression and DNA copy number were determined in parallel from the same replicate culture. The underlying data for all panels can be found in S1 Data.
