# Supplementary material for: Low levels of tetracyclines select for a mutation that prevents the evolution of high-level resistance to tigecycline
Source: PLoS Biol. 2022 Sep 28;20(9):e3001808. doi: 10.1371/journal.pbio.3001808 (PMC9550176; doi:10.1371/journal.pbio.3001808)
Supplement: S3 Fig — (PDF) [file pbio.3001808.s015.pdf]

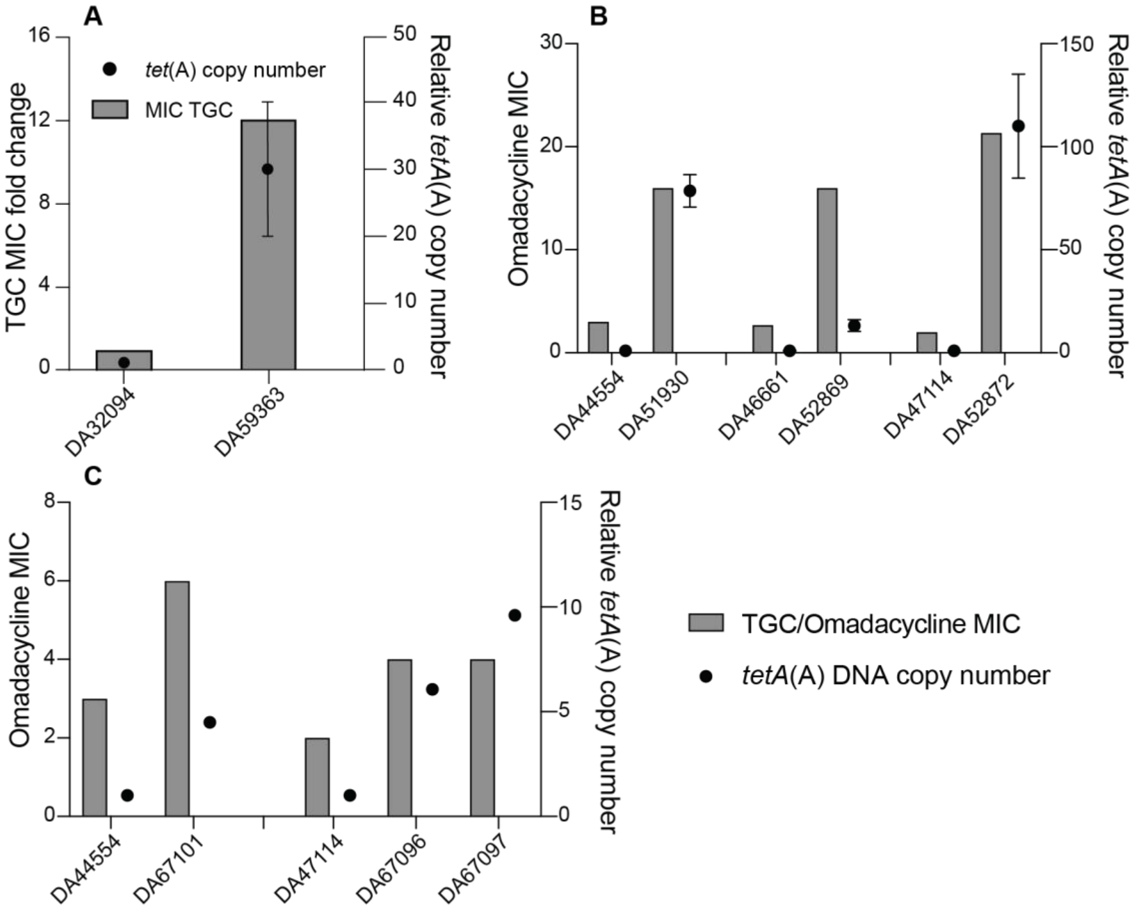

S3 Fig. Effect of *tet*(A)*wt* amplifications on TGC MIC in a clinical *Klebsiella pneumoniae* and omadacycline MICs in *E. coli.* **A**. Increased TGC resistance linked to *tet*(A)*wt* amplification in *Klebsiella pneumoniae*. DA59363 is a spontaneous TGC-resistant mutant of DA32094. *tet*(A)*wt* copy number was determined by qPCR and is the result of 3 independent measurements. **B.** Omadacycline MICs and DNA copy number of clinical *E. coli* isolates carrying *tet*(A)*wt* (DA44554, DA46661, DA47114) and TGC resistant mutants with amplifications of *tet*(A)*wt* (DA51930, DA52869, DA52872 respectively). MICs and DNA copy numbers determined from three biological replicates, standard deviation shown. **C.** Omadacycline MICs and DNA copy number of clinical isolates carrying *tet*(A)*wt* (DA44554, and DA47114) and mutants selected in presence of 8 mg/L omadacycline (DA67101, DA67096, and DA67097). MICs and DNA copy numbers determined in parallel on same cultures to minimize the loss of amplifications and therefore without replicates for those mutants. MICs for parental isolates determined from three biological replicates. The underlying data for all panels can be found in S1 Data.
