# Supplementary material for: Low levels of tetracyclines select for a mutation that prevents the evolution of high-level resistance to tigecycline
Source: PLoS Biol. 2022 Sep 28;20(9):e3001808. doi: 10.1371/journal.pbio.3001808 (PMC9550176; doi:10.1371/journal.pbio.3001808)
Supplement: S4 Fig — (PDF) [file pbio.3001808.s016.pdf]

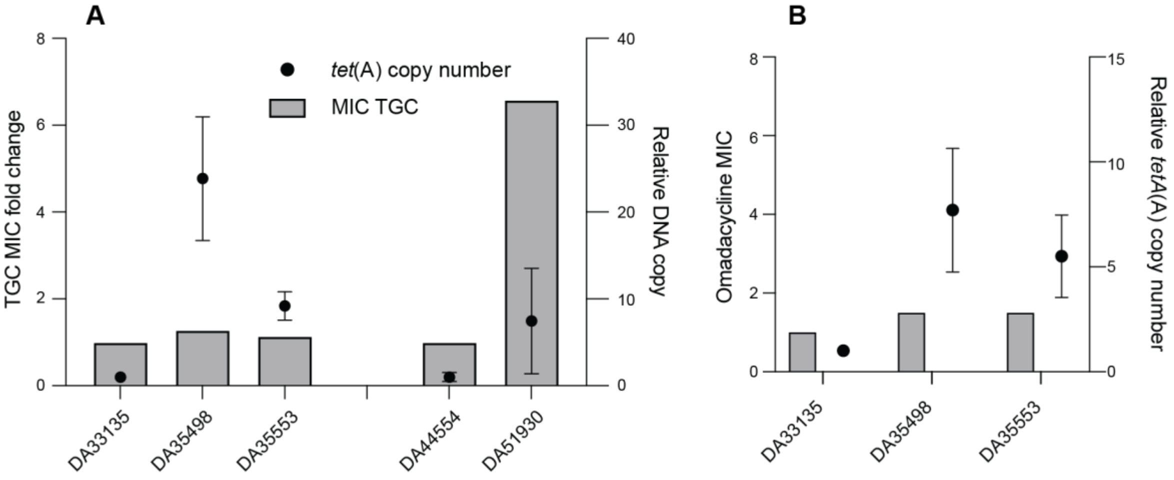

S4 Fig. Effect of *tet*(A)$^{\Delta tetR}$ amplification on TGC and omadacycline MIC. **A.** DA35498 and DA35553 are mutants of DA33135 that carry amplifications of *tet*(A)$^{\Delta tetR}$. DA51930 is a mutant of DA44554 that carries an amplification of *tet*(A)^{wt} and is used here as a reference to show the effect on TGC MIC of *tet*(A)^{wt} amplification. Relative DNA copy numbers of *tet*(A) are normalized to chromosomal control. **B.** Omadacycline MICs and DNA copy number of a clinical isolate carrying *tet*(A)$^{\Delta tetR}$ (DA33135) and mutants carrying amplifications of the region containing *tet*(A)$^{\Delta tetR}$ (DA35498 and DA35553) [10]. MICs (median) and DNA copy numbers (mean) determined from three biological replicates, standard deviation shown. The underlying data for both panels can be found in S1 Data.
