# Supplementary material for: Low levels of tetracyclines select for a mutation that prevents the evolution of high-level resistance to tigecycline
Source: PLoS Biol. 2022 Sep 28;20(9):e3001808. doi: 10.1371/journal.pbio.3001808 (PMC9550176; doi:10.1371/journal.pbio.3001808)
Supplement: S5 Fig — (PDF) [file pbio.3001808.s017.pdf]

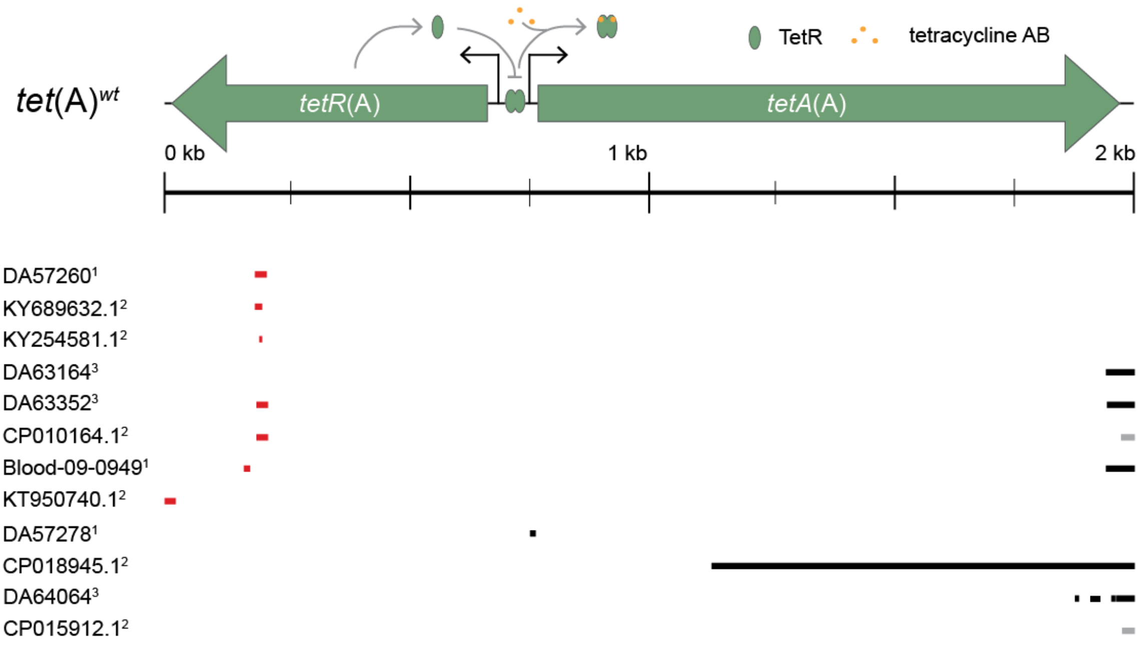

S5 Fig. Schematic of all observed *tet*(A) alleles. For each allele, one example of each variant found is indicated. Isolates are from the three collections: 1) NCBI, 2) UPEC and blood *E. coli*, and 3) in-house bacteremia *E. coli* isolates. Deletions affecting *tetR*(A) and *tetA*(A) are shown in red and black, respectively. Gray lines show disruptions immediately downstream of *tetA*(A), potentially affecting termination of transcription. Dashed lines represent the range in which the deletion may start, as the exact starting point is unknown.
