# Supplementary material for: Low levels of tetracyclines select for a mutation that prevents the evolution of high-level resistance to tigecycline
Source: PLoS Biol. 2022 Sep 28;20(9):e3001808. doi: 10.1371/journal.pbio.3001808 (PMC9550176; doi:10.1371/journal.pbio.3001808)
Supplement: S6 Fig — (PDF) [file pbio.3001808.s018.pdf]

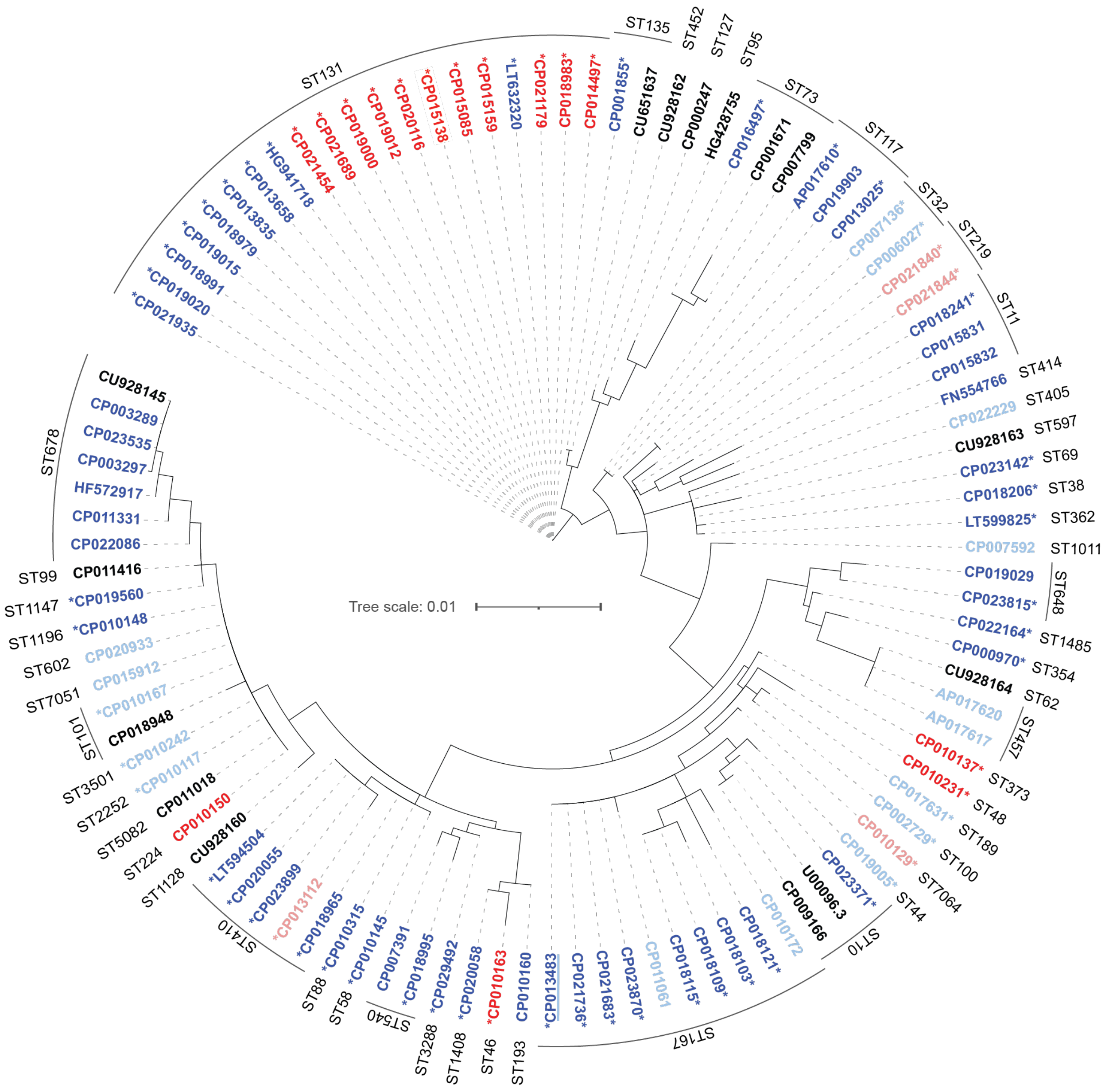

S6 Fig. Phylogeny of *tet*(A)-carrying isolates from NCBI. Maximum likelihood phylogeny based on MLST alleles of *tet*(A)-carrying isolates from NCBI, where a complete genome was available, with isolates from S1 Table included for comparison (in black). Accession numbers for chromosomes of isolates shown in figure. Dark blue: wild-type *tet*(A). Light blue: 28 bp truncation of *tetA*(A). Dark red: *tet*(A)$^{\Delta tetR}$ (with the 24-bp deletion in *tetR*(A)). Light red: other deletion in *tetR*(A) (6- or 15-bp deletion, in same site as 24-bp deletion). Occasional isolates carried several *tet*(A), with CP013483 carrying two different *tet*(A) determinants, as indicated with the shades of blue colours used. Occasional point mutations were sometimes also present in *tet*(A) but are not specified here. Accession numbers marked with an asterisk represent isolates where *tet*(A) was located on a plasmid, with all other isolates carrying *tet*(A) on the chromosome.
