# Supplementary material for: Low levels of tetracyclines select for a mutation that prevents the evolution of high-level resistance to tigecycline
Source: PLoS Biol. 2022 Sep 28;20(9):e3001808. doi: 10.1371/journal.pbio.3001808 (PMC9550176; doi:10.1371/journal.pbio.3001808)
Supplement: S7 Fig — (PDF) [file pbio.3001808.s019.pdf]

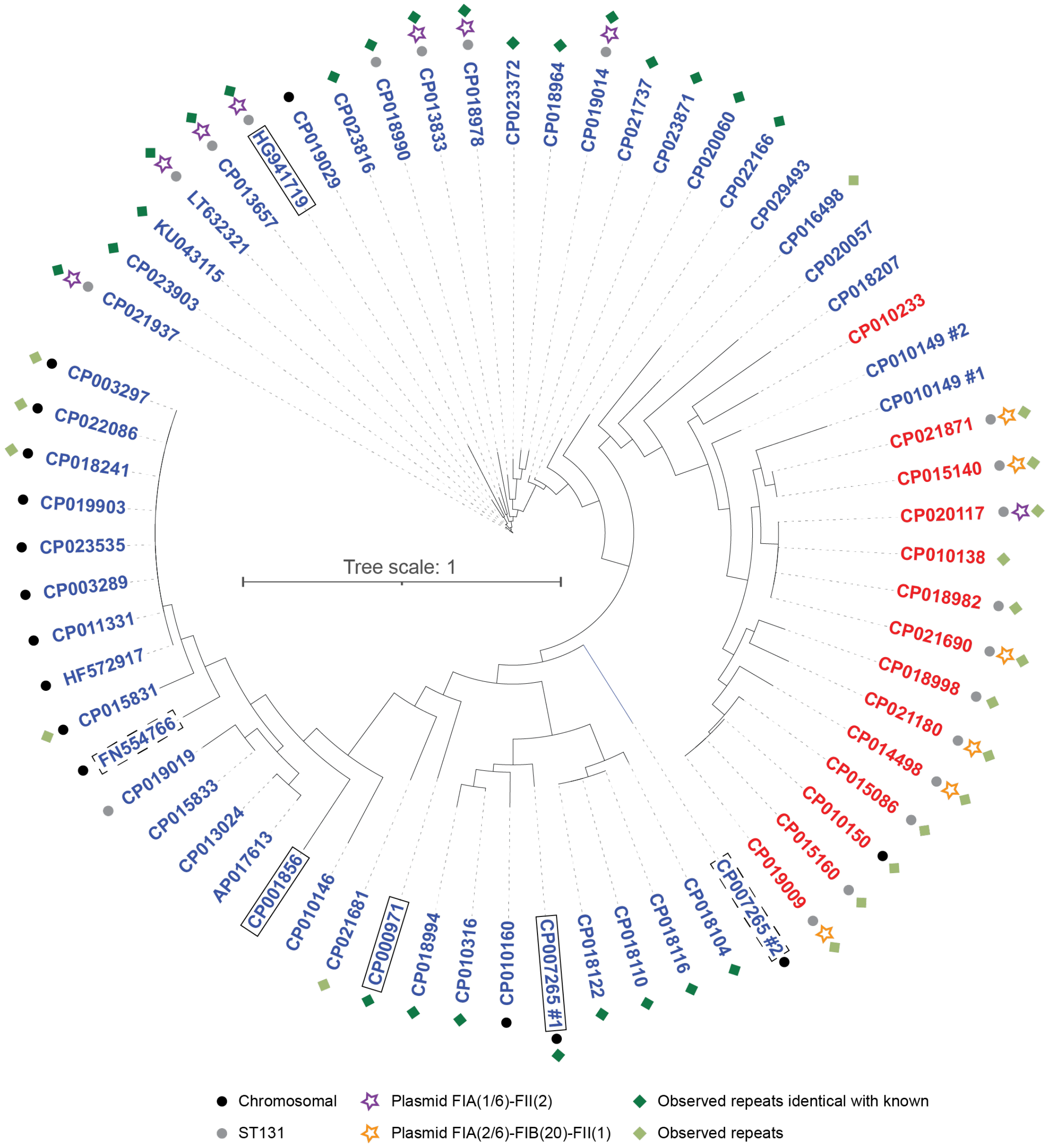

S7 Fig. Phylogeny of genetic context surrounding *tet*(A) in isolates from NCBI. Maximum likelihood phylogeny based on 7-kb regions upstream and downstream of *tet*(A)-carrying isolates from NCBI, including only *tet*(A)^{wt} and *tet*(A)$^{\Delta tetR}$ with no other mutations. Accession numbers for chromosomes of isolates shown in figure. Blue text: wild-type *tet*(A). Red text: *tet*(A)$^{\Delta tetR}$ (with the 24-bp deletion in *tetR*(A)). Isolates with boxed accession numbers were included in our screen. Solid box: amplifications of *tet*(A) were observed. Dashed box: no amplifications were observed. Numbers after accession number indicate alleles observed on same isolate. Unless indicated otherwise, *tet*(A) determinants were located on plasmids. Specific frequently observed plasmids are indicated by coloured stars. Cases with large identical repeats present in the same orientation on each side of *tet*(A) are indicated with green diamonds, with dark green corresponding to repeats involved in *tet*(A)^{wt} amplifications in TGC resistant mutants of isolates included in our screen. Light green diamonds correspond to other observed repeats (458 to 820 bp in length) that could hypothetically allow for *tet*(A) amplification.
