# Supplementary material for: Low levels of tetracyclines select for a mutation that prevents the evolution of high-level resistance to tigecycline
Source: PLoS Biol. 2022 Sep 28;20(9):e3001808. doi: 10.1371/journal.pbio.3001808 (PMC9550176; doi:10.1371/journal.pbio.3001808)
Supplement: S8 Fig — (PDF) [file pbio.3001808.s020.pdf]

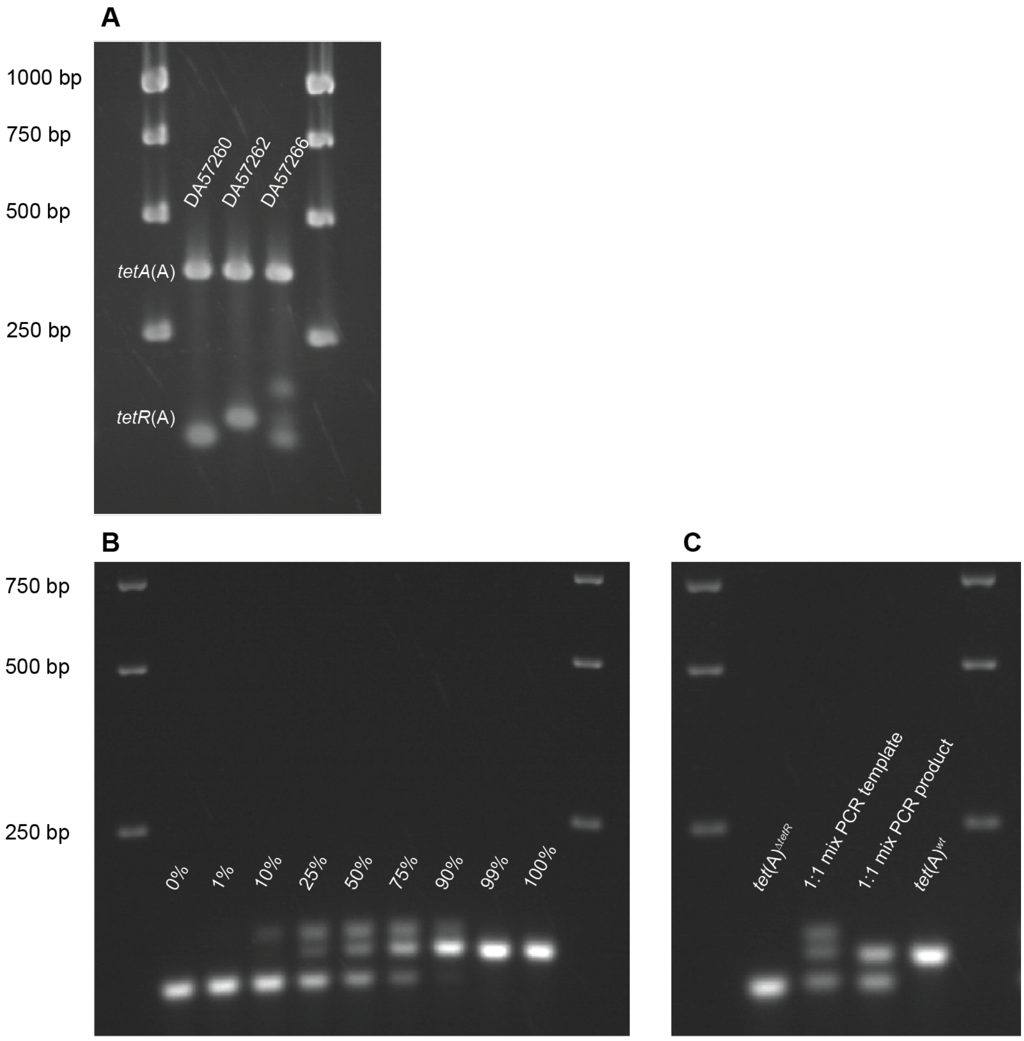

S8 Fig. PCR screen to detect presence of *tet*(A) and differentiate the *tet*(A) alleles. A. The PCR screen can identify the presence of *tet*(A) (upper band, 375 bp) and whether the *tetR*(A) is wild-type (DA57262, lower band, 130 bp) or carries the 24-bp deletion (DA57260, lower band, 105 bp). The screen can also identify if both alleles are present (DA57266). **B.** PCR of *tetR*(A) only, where *tet*(A)^{wt} and *tet*(A)$^{\Delta tetR}$ template have been mixed before PCR, showing percentage of *tet*(A)^{wt} in mix from 0 to 100%. **C.** 1:1 mixing of template of *tet*(A)^{wt} and *tet*(A)$^{\Delta tetR}$ before PCR compared to mixing of product after PCR, revealing that the unexpected band pattern observed for DA57266 in panel A) is a result of the PCR reaction.
