# Supplementary material for: Low levels of tetracyclines select for a mutation that prevents the evolution of high-level resistance to tigecycline
Source: PLoS Biol. 2022 Sep 28;20(9):e3001808. doi: 10.1371/journal.pbio.3001808 (PMC9550176; doi:10.1371/journal.pbio.3001808)
Supplement: S9 Fig — (PDF) [file pbio.3001808.s021.pdf]

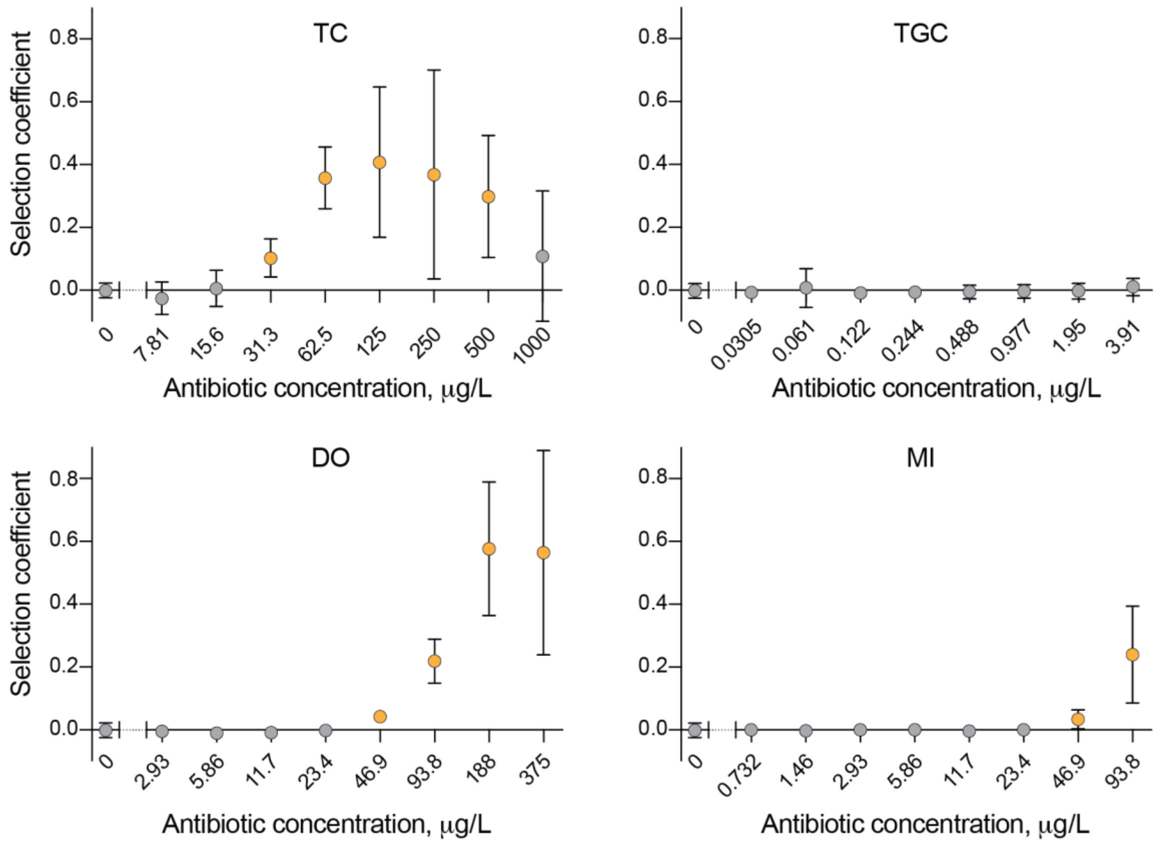

S9 Fig. Competition of a strain carrying *tet*(A)$^{\Delta tetR}$ *vs* an isogenic strain without any *tet*(A) allele at different antibiotic concentrations. TC: tetracycline; TGC: tigecycline; DO: doxycycline; MI: minocycline. Selection coefficients show *tet*(A)$^{\Delta tetR}$ over no *tet*(A). The highest concentration of TC analysed (1/32- times MIC of strain carrying *tet*(A)$^{\Delta tetR}$; MIC: 0.75-1.0 mg/L) is at the MIC of *E. coli* without *tet*(A). Growth of the strains without *tet*(A) is largely inhibited at this concentration, allowing for out-competition by mutants that arose during the experiment. This leads to very high variation at this concentration for TC and an inaccurate measurement of fitness cost. Orange circles: *tet*(A)$^{\Delta tetR}$ has a significant fitness advantage. Grey circles: neither allele has a significant fitness advantage. The underlying data can be found in S1 Data.
