# Supplementary material for: Low levels of tetracyclines select for a mutation that prevents the evolution of high-level resistance to tigecycline
Source: PLoS Biol. 2022 Sep 28;20(9):e3001808. doi: 10.1371/journal.pbio.3001808 (PMC9550176; doi:10.1371/journal.pbio.3001808)
Supplement: S10 Fig — (PDF) [file pbio.3001808.s022.pdf]

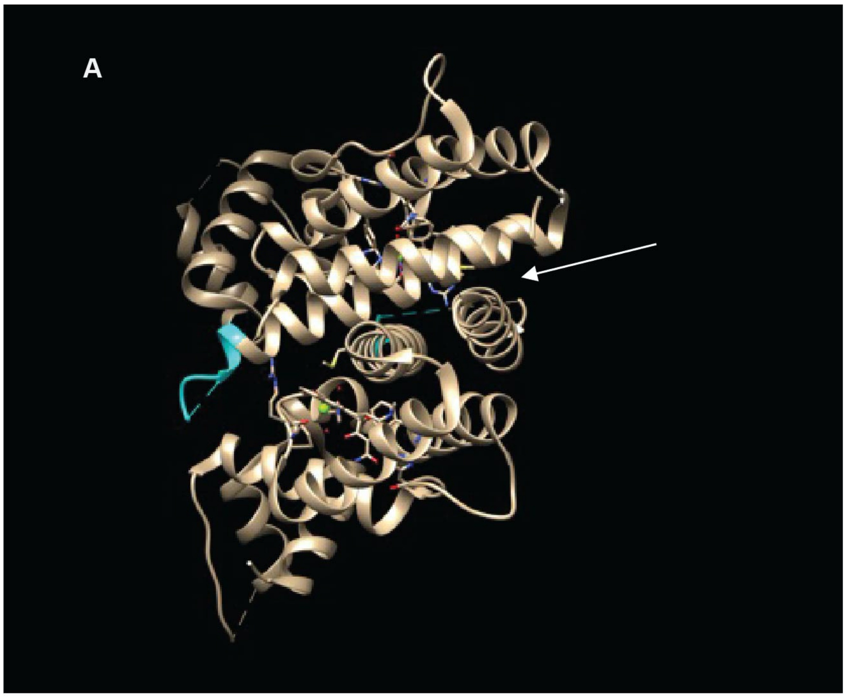

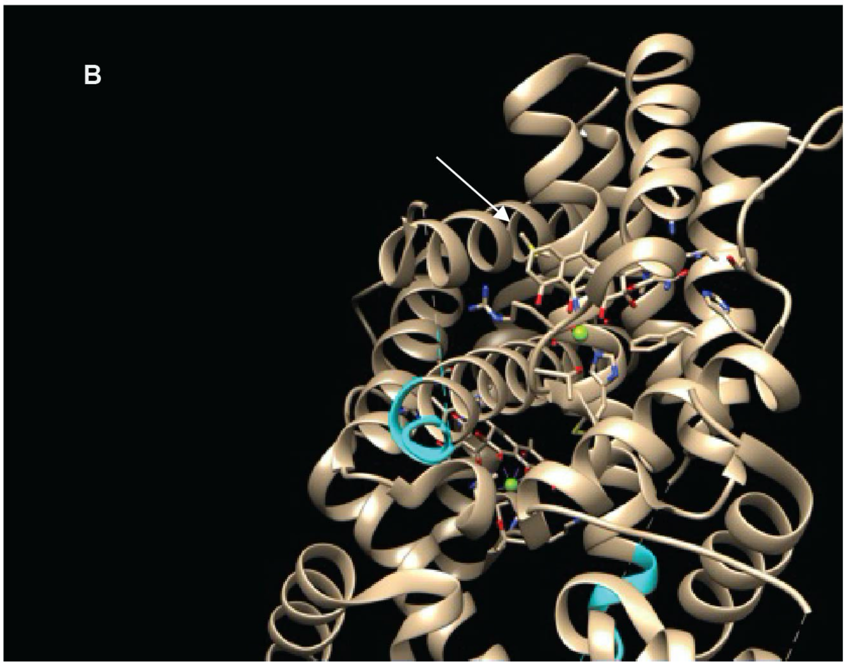

S10 Fig. Protein structure and site of 8 amino-acid deletion in TetR(A). TetR(A)^{wt} dimer, with tetracycline. Structure analyzed in UCSF Chimera, from PDBe reference 5mru. Deletion region in TetR(A)$^{8aa\Delta}$ labeled cyan. **A.** Dimer showing proximity to dimerization face (emphasized with white arrow). **B.** Dimer showing proximity to C9 tetracycline residue (white arrow).
