# Supplementary material for: Low levels of tetracyclines select for a mutation that prevents the evolution of high-level resistance to tigecycline
Source: PLoS Biol. 2022 Sep 28;20(9):e3001808. doi: 10.1371/journal.pbio.3001808 (PMC9550176; doi:10.1371/journal.pbio.3001808)
Supplement: S11 Fig — (PDF) [file pbio.3001808.s023.pdf]

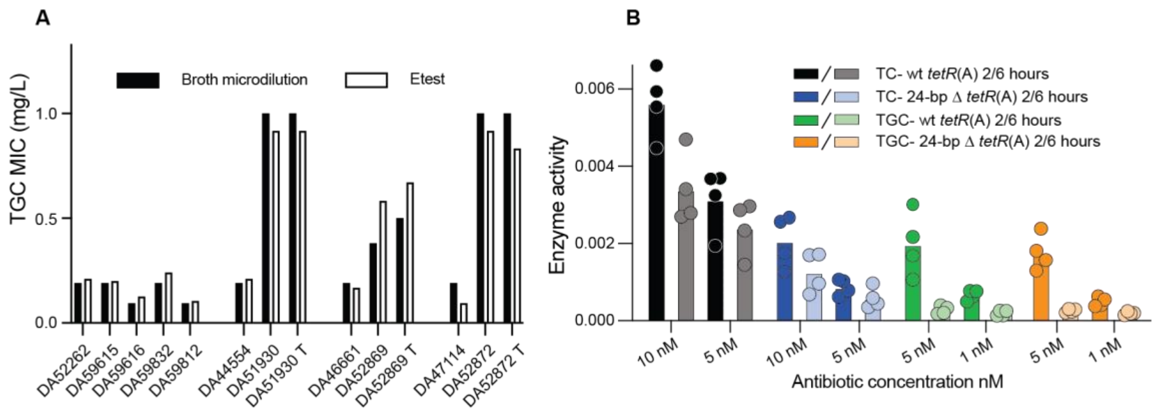

S11 Fig. Method control experiments A. MICs as measured using broth microdilution or Etest strips. DA51930, DA52869 and DA52872 are TGC resistant mutants of DA44554, DA46661 and DA47114, respectively. T: MICs determined under constant selective pressure to maintain the *tet*(A)^{wt} amplifications (presence of 0.25 mg/L TGC). **B.** β-galactosidase assay diffusion rate comparisons. The relative enzyme activity of *lacZ* was compared for TC (black and blue) and TGC (green and orange) at 2 and 6 hours (dark and light color, respectively) for constructs regulated by either *tetR*(A)^{wt} or *tetR*(A) carrying the 24-bp deletion present in the *tet*(A)$^{\Delta tetR}$ allele. The underlying data can be found in S1 Data.
