# Supplementary material for: Low levels of tetracyclines select for a mutation that prevents the evolution of high-level resistance to tigecycline
Source: PLoS Biol. 2022 Sep 28;20(9):e3001808. doi: 10.1371/journal.pbio.3001808 (PMC9550176; doi:10.1371/journal.pbio.3001808)
Supplement: S1 Results — (PDF) [file pbio.3001808.s024.pdf]

S1 Result. Association of *tetR*(A) mutation with ST131 group

Since the *tet*(A)$^{\Delta tetR}$ allele was common, we used the sequenced *tet*(A)-carrying *E. coli* isolates from NCBI where a complete genome was available and the UPEC and blood *E. coli* isolate collection [1] to determine if clonal spread of that allele could, in part, account for its high occurrence. For the UPEC and blood *E. coli* isolate collection, 31.6% (12/38) of the isolates carrying *tet*(A) (any allele) belonged to ST131, and 63.3 % (9/14) of the isolates that carried the *tet*(A)$^{\Delta tetR}$ allele were of the ST131 sequence type. The proportion of ST131 isolates in the entire study was 15.4% (40/259). The probability of an isolate carrying the *tet*(A)$^{\Delta tetR}$ allele to be an ST131 clone was higher than the probability of all isolates in the study to belong to ST131 (two-proportion z-test with Yates continuity correction, $p<0.0001$). Isolates in other ST-groups also carried the *tet*(A)$^{\Delta tetR}$ allele. A phylogenetic tree of the isolates in this collection can be found in Fig 1B of the original publication [1].

The ST-group was determined for the sequenced *tet*(A)-carrying *E. coli* isolates from NCBI where a complete genome was available (n=95). 22.1% (21/95) of the isolates carrying *tet*(A) (any allele) belonged to ST131, and 70.6 % (12/17) of the isolates that carried the *tet*(A)$^{\Delta tetR}$ allele were of the ST131 sequence type. The probability of an isolate carrying the *tet*(A)$^{\Delta tetR}$ allele to be an ST131 clone was higher than the probability of all *tet*(A)-carrying isolates in the collection to belong to ST131 (two-proportion z-test with Yates continuity correction, $p<0.001$) (S8 Table). A phylogenetic tree of the isolates in this collection can be found in S6 Fig.

Plasmid typing was performed for the sequenced *tet*(A)-carrying *E. coli* isolates from NCBI genome when a complete genome was available (n=95). *tet*(A) was observed on a variety of plasmid types, although primarily on IncF plasmids (S8 Table). In the ST131 isolates, *tet*(A)$^{\Delta tetR}$ from 11 isolates analysed was present on IncF plasmids of five different plasmid alleles types, while *tet*(A)^{wt} from 9 isolates analysed was present on IncF plasmids of two different plasmids alleles types, and on one IncHI2 plasmid. Within ST131 isolates, plasmid types FIA(1/6)- FII(2) (8 ST131 isolates in total, *tet*(A)^{wt}=7, *tet*(A)$^{\Delta tetR}$=1) and FIA(2/6)-FIB(20)-FII(1) (6 ST131 isolates in total, all *tet*(A)$^{\Delta tetR}$) were the most common (S7 Fig, S8 Table).

References

1. Salipante SJ, Roach DJ, Kitzman JO, Snyder MW, Stackhouse B, Butler-Wu SM, et al. Large-scale genomic sequencing of extraintestinal pathogenic *Escherichia coli* strains. Genome Res. 2015 Jan;25(1):119–28.
