# Supplementary material for: Low levels of tetracyclines select for a mutation that prevents the evolution of high-level resistance to tigecycline
Source: PLoS Biol. 2022 Sep 28;20(9):e3001808. doi: 10.1371/journal.pbio.3001808 (PMC9550176; doi:10.1371/journal.pbio.3001808)
Supplement: S2 Results — (PDF) [file pbio.3001808.s025.pdf]

S2 Result. Optimization of multiplex PCR screen

The *tet*(A)^{wt} allele cannot be easily distinguished from *tet*(A)$^{\Delta tetR}$ purely on phenotypic observations (see S6 Table). To generate a screening procedure to identify *tet*(A)^{wt} and *tet*(A)$^{\Delta tetR}$, we optimized a simple multiplexed PCR screen that both detects presence of *tet*(A) and identifies the *tetR*(A) allele. The high similarities present in *tetR*(A) sequence at and around the 24-bp deleted region did not allow us to design PCR primers that could efficiently amplify only one of the two alleles. Instead, size-discrimination of the PCR product amplifying part of *tetR*(A) was used to distinguish the different *tet*(A) alleles. The multiplex PCR was tested on 22 whole-genome sequenced isolates (13 with *tet*(A) from a collection of UPEC and blood isolates [1] and 9 without *tet*(A) from previously screened isolates, included in S2 Table) and successfully identified all *tet*(A)-carrying strains, as well as the correct *tet*(A) allele (subset shown in S8A Fig). Multiplex *tet*(A) alleles can be present in an isolate but a single *tet*(A)^{wt} copy can allow for spontaneous, clinically-relevant TGC resistance to develop. We showed that a *tet*(A)^{wt} allele present at ≥10% of all copies of *tet*(A) could be correctly detected in our PCR screen, which also detected the presence of both alleles in one *E. coli* isolate (S8B and S8C Fig).

References

1. Salipante SJ, Roach DJ, Kitzman JO, Snyder MW, Stackhouse B, Butler-Wu SM, et al. Large-scale genomic sequencing of extraintestinal pathogenic *Escherichia coli* strains. Genome Res. 2015 Jan;25(1):119–28.
