# Supplementary material for: Low levels of tetracyclines select for a mutation that prevents the evolution of high-level resistance to tigecycline
Source: PLoS Biol. 2022 Sep 28;20(9):e3001808. doi: 10.1371/journal.pbio.3001808 (PMC9550176; doi:10.1371/journal.pbio.3001808)
Supplement: S1 Method — (PDF) [file pbio.3001808.s026.pdf]

S1 Method. Stepwise selection for increased TGC resistance

A set of strains with high mutant frequencies (all carrying wild-type *tet*(A): DA44554, DA52262, DA47114, DA50061, DA45430, DA46661, and DA52394) were subjected to stepwise mutant selection at increasing TGC concentrations, with two concentrations run in parallel per selection round (4 mg/L and 8 mg/L, 12 mg/L and 16 mg/L, and 24 mg/L and 32 mg/L TGC). For DA44554, larger increases in TGC selection concentrations were also tested (see S4 Table and S1 Fig). Selections were performed as described in main text under "Simplified fluctuation assay". If mutants were only found on the lower of the two concentrations tested (for example at TGC 4 mg/L but not at 8 mg/L) those mutants were subsequently subjected to selection at only the higher of the previous paired concentrations at the next round (for example 8 mg/L). Once mutants could no longer be selected on higher TGC concentration, selection was stopped for that strain. MICs were performed for 2 to 8 mutants per selection step, as described in main text under "MIC determinations".
