# Supplementary material for: Low levels of tetracyclines select for a mutation that prevents the evolution of high-level resistance to tigecycline
Source: PLoS Biol. 2022 Sep 28;20(9):e3001808. doi: 10.1371/journal.pbio.3001808 (PMC9550176; doi:10.1371/journal.pbio.3001808)
Supplement: S2 Method — (PDF) [file pbio.3001808.s027.pdf]

S2 Method. TGC MIC and fitness cost with overexpression of *tetA*(A)

pBad30 plasmids, carrying *tetA*(A) behind an arabinose-inducible promoter or empty [1], were electroporated into an ara$^-$ MG1655 (DA73831 and DA73834, respectively) and transformants were selected in presence of 100 mg/L ampicillin. Three biological replicates each of DA73831 and DA73834 were inoculated in 1 ml MHB supplemented with 100 mg/L ampicillin and incubated overnight at 37˚C. TGC MIC was then determined using Etests as described above with MHA plates supplemented with 0, 0.0125%, 0.025%, 0.05%, 0.75%, 0.1%, 0.2%, 0.3%, or 0.4% arabinose. While tryptone broth is better adapted to arabinose induction than MHB, TGC MICs on tryptone broth plates were incorrect and that medium could not be used for this experiment.

Comparative growth rates for each strain were determined using a Bioscreen C (Oy Growth Curves AB Ltd). Three biological replicates each of DA73831 and DA73834 were inoculated (1 ul in 1 mL) in tryptone broth (1% tryptone, 0.5% NaCl) supplemented with 100 mg/L ampicillin and 0, 0.0004883%, 0.0009768%, 0.001954%, 0.003906%, 0.007813%, 0.01563%, 0.03125%, 0.0625%, 0.1255%, 0.25% arabinose respectively. Three technical replicates of 200 ul were used for each strain and condition. Comparative growth rates were measured over 9 hours at 37 ˚C and determined by the rise of the slope during exponential phase (OD600 0.02 to 0.05).

References

1. Linkevicius M, Sandegren L, Andersson DI. Potential of tetracycline resistance proteins to evolve tigecycline resistance. Antimicrob Agents Chemother. 2016 Jan 29;60(2):789–96.
