# Supplementary material for: Low levels of tetracyclines select for a mutation that prevents the evolution of high-level resistance to tigecycline
Source: PLoS Biol. 2022 Sep 28;20(9):e3001808. doi: 10.1371/journal.pbio.3001808 (PMC9550176; doi:10.1371/journal.pbio.3001808)
Supplement: S3 Method — (PDF) [file pbio.3001808.s028.pdf]

S3 Method. Comparison of Etests and broth microdilution MIC determination

Broth microdilutions were performed using fresh MHB with TGC concentrations ranging from 1.0 mg/L down to 0.024 mg/L (media serially two-fold diluted from starting concentrations of 1.0 and 0.75 mg/L to obtain a similar range of TGC concentrations as present on Etest strips) in 96-well plates. Each well (containing 100 µl of media with different amounts of TGC) was inoculated with approximately 1-5 x 10^5 cells from overnight culture. For omadacycline, oxytetracycline, and chlortetracycline, MICs determined using broth microdilution inoculated with 1-5 x 10^4 cells. Biological triplicates were used, and plates were covered from light and incubated at 37˚C with shaking at 190 rpm for 18 hours before visual readings according to EUCAST guidelines [1] and OD_{620nm} measurement using a Multiskan FC plate reader (Thermo Scientific). Etests were performed in parallel as described in main text under "MIC determinations" for each replicate and MICs were determined to be slightly variable but largely comparable (S11A Fig). Therefore, Etests were deemed useable throughout this study to determine TGC MICs.

References

1. EUCAST. EUCAST reading guide for broth microdilution [Internet]. 2019. Available from: http://www.eucast.org/fileadmin/src/media/PDFs/EUCAST_files/Disk_test_documents/2019_manuals/Reading_guide_BMD_v_1.0_2019.pdf
