# Supplementary material for: Low levels of tetracyclines select for a mutation that prevents the evolution of high-level resistance to tigecycline
Source: PLoS Biol. 2022 Sep 28;20(9):e3001808. doi: 10.1371/journal.pbio.3001808 (PMC9550176; doi:10.1371/journal.pbio.3001808)
Supplement: Attachment — Submitted filename: Response_reviewers_revision.pdf [file pbio.3001808.s030.pdf]

Clonal spread and antibiotic selection of a mutation preventing the evolution of high-level resistance

Jennifer Jagdmann, Dan I. Andersson & Hervé Nicoloff

Response to reviewers

Dear Editor,

Thank you for the thorough feedback regarding our manuscript. The questions and comments have been addressed and the manuscript amended according to the suggestions of you and the reviewers. Please find here under answers to each of your reviewing points. The changes to the manuscript and supplemental data have been tracked in one of the files submitted. The quality of the manuscript and figures has been clearly improved thanks to your feedback and the reviewer comments. Some of the reviewers' remarks led us to perform additional analyses, which have greatly strengthened our observation of clonal spread of the mutation in *tetR*(A). Because of these additional analysis, we have now modified the title of the manuscript and adjusted the summary (Lines 34-36 and 38-39) to better acknowledge the importance of both clonal spread and antibiotic selection in the frequent occurrence of the mutation preventing the evolution of high-level resistance among *E. coli* isolates. We hope that the manuscript is now in an acceptable form.

If you have any further questions or suggestions, do not hesitate to contact me.

Sincerely,
Hervé Nicoloff

Reviewer #1:

1. Results, subheading identification of TGC resistance mechanisms. Were basal MIC homogeneous? Any impact of basal MIC on MF or resistance development? Did tet(A) positive show higher basal MICs?

Basal tigecycline MICs were not homogenous, as can be seen in Table S2 (other tetracyclines can be found in Table S6, for a subset). There is a weak positive correlation between parental MIC and mutant frequency at 0.5 mg/L tigecycline ($R^2 = 0.3168$). As observed in Table S6, while parental TGC MICs for *tet*(A)^{wt} positive strains are often higher (one or two Etest increments higher, within the range of error for an Etest) than MICs for *tet*(A)$^{\Delta tetR}$-carrying strains or strains without *tet*(A), there is an overlap between these groups that impedes differentiation by MIC. The slightly elevated MIC of *tet*(A)^{wt} positive strains does therefore not explain the frequent resistance development observed with strains carrying *tet*(A)^{wt}. We have extended this in our text as well (lines 177-181).

2. Lines 127-150. Was gene dosage amplification the only mechanism leading to increased expression? Correlation with transcription data?

Yes it was and we did not observe any mutations in the promotor region of *tetA*(A). We have added an mRNA expression analysis confirming the increase in transcription with increased

gene dosage (Lines 142-143, Figure S2C).

3. Lines 183-186. Was tet(A) expression similar in tetR wt and delta evolved strains in addition to having similar gene dosages?

We have added an mRNA expression analysis comparing mRNA transcription with gene dosage for *tet*(A)$^{\Delta tetR}$ as well (Lines 196-197, Figure S2C). We did not see an increase in mRNA expression with induction for the amplified *tet*(A)$^{\Delta tetR}$-carrying isolate, but did see a similar small increase in leaky expression with amplifications without induction as for the *tet*(A)^{wt} -carrying isolate (approximately 2x leaky expression without induction for both).

4. Not fully clear what is the effect of a fully truncated tetR on tetA basal or induced expression as compared with delta 24.

We did not study any fully truncated *tetR*(A)-carrying isolates in our study, nor were any observed in our work but we would expect *tetA*(A) to be fully induced since the repressor would be gone. Our analysis of *tetA*(A) under the control of an arabinose-induced promotor instead of *tetR*(A) (Figure S2) shows that gradual induced expression (in presence of increasing amounts of arabinose) increases MIC and fitness cost.

5. If tetR delta 24 fails to increase tetA expression in the presence of TGC should this not have an impact on susceptibility/resistance to the drug?

While *tet*(A)$^{\Delta tetR}$-carrying strains, with decreased *tetA*(A) expression, do often have lower TGC MICs than *tet*(A)^{wt}-carrying strains (see Table S6), the TetA(A) pump itself is not very effective at effluxing tigecycline (as observed by the minimal increase in MIC observed in Table S6 compared to isolates without *tet*(A)), causing a difficult-to-distinguish difference in MIC between *tet*(A)^{wt}- and *tet*(A)$^{\Delta tetR}$-carrying strains.

6. Lines 337-338. Why not TGC?

Minocycline was used as a selection pressure for *tet*(A)$^{\Delta tetR}$ as it gave the greatest observed growth advantage of *tet*(A)$^{\Delta tetR}$ compared to *tet*(A)^{wt}. *tet*(A)$^{\Delta tetR}$ had a lower observed growth advantage at the tested concentrations of TGC. We have modified the introduction to this part of the results to better clarify the reasoning behind the experiment (Lines 370-374).

Reviewer #2:

1. Line 70-71. Please clarify what 'moderate reduction' means quantitatively. Similarly, please report the clinical breakpoint for TGC and the quantitative definition of "high TGC MICs" and "high-level TGC resistance". These appear in the 'Discussion' section, but it would be helpful to have in the introduction.

Precise MICs and resistance levels have been added in the text, to lines 72-73 as well as 75-76. Clinical resistance breakpoints have been added as well.

2. Lines 71-73. Are TGC resistance mutations described in other Enterobacteriales? On a quick perusal of the literature, it looks like TGC treatment failure due to tet(A) mutations has been described in K. pneumoniae (e.g., PMID 33717043).

Clarifying text has been added to line 69. However, this study has focused on *E. coli*, with the exception of confirming the resistance mechanism via amplifications in *K. pneumoniae*, and therefore focuses on TGC resistance in *E. coli*.

3. For the isolates with tet(A), were all the sequences of tet(A) identical? For the 53 tet(A) isolates used in the screen, could you provide a phylogeny based only on the tet(A) sequence so we could understand how similar they are? Or were all those marked as 'wt' actually identical? Similarly, are those with the 24bp del in tetR(A) otherwise genetically identical?

Not all 53 isolates in the screen (i.e., in Table S2) carried *tet*(A), and not all were sequenced (see Table S10). Isolates from the blood isolate collection, isolates classed as TGC resistant in clinical laboratory, and isolate DA33131 were not sequenced, rather *tet*(A) presence and allele was determined by PCR. However, the majority of the isolates in the screen were sequenced, and in these isolates, all *tet*(A)^{wt} were identical and all *tet*(A)$^{\Delta tetR}$ were identical, with no point mutations observed. We have clarified which isolates were not sequenced in the footnotes of Table S2.

4. In the isolates with amplification of tet(A), were the copies exact, or were there mutations in the amplified tet(A)?

We did not observe any mutations in amplified *tet*(A). However, due to the multiple copies of *tet*(A) when amplified, if present at low frequency (e.g., if one copy of *tet*(A) is mutated while many other copies remain wild-type), our sequencing would not allow us to detect such mutations with enough accuracy.

Amplifications can be an evolutionary intermediate steps towards *tet*(A) evolution to increase efflux efficiency (since multiple *tet*(A) copies would increase mutation target size and the likelihood of acquiring beneficial mutations in *tet*(A)). This could ultimately lead to reversion of amplifications while maintaining high TGC MIC due to mutations in the evolved *tet*(A). This has been shown in other systems but is was not the focus of this study.

5. Lines 136-8. While overexpression can lead to increased TGC MIC, to show that this explains that the amplifications were associated with increased tet(A) dosage, do you have transcriptional data showing that there was in fact increased tet(A) expression in the clinical isolates?

We have added an mRNA expression analysis confirming the increase in transcription with increased gene dosage in a clinical isolate with *tet*(A)^{wt} (Lines 142-143, Figure S2C).

6. Figure S1. What does 'amplification 9.5x' refer to (at about 6 o'clock on the circle in the figure)? In what way is there a half of an amplification?

WGS or qPCR gives the average copy number per cell in the population, that can lead to any number.

7. Lines 159-161. For the K. pneumoniae experiments, were the amplifications sequenced or only counted by PCR?

Amplifications were quantified by qPCR. WGS was not performed on *K. pneumoniae*. This is now specified in the text (Line 166).

8. Lines 161-166. Please indicate in the text and/or on Fig S4 the breakpoint for omadacycline resistance.

There is no CLSI or EUCAST *E. coli* clinical breakpoint for omadacycline to date.

9. Section about prevalence. I'm confused about this '200' isolates in the NCBI E. coli genome sequences, as my quick check suggests nearly 30,000 genome sequences. https://www.ncbi.nlm.nih.gov/genome/browse/#!/prokaryotes/167/ Where were the 200 from? Please provide a link. For collections ii and iii as sited, it'd be really helpful to see all of these on a phylogeny to understand whether the instances of tet(A) represent clonal expansion or multiple occurrences in distinct lineages.

The 200 isolates in the NCBI *E. coli* sequences carrying *tet*(A) were identified by BLAST, limiting the search to complete *E. coli* sequences (as was possible at the time, in 2016-2017), with *tetA*(A) as the query. This resulted in complete WGS *E. coli* and sequenced plasmids that were labeled as "complete" in *E. coli* and carried *tet*(A). A similar search as the one described in the comment here, limiting results to "complete", results in 2278 complete *E. coli* genomes potentially carrying *tet*(A) to date (https://www.ncbi.nlm.nih.gov/genome/browse/#!/prokaryotes/167/). We have clarified this in the Results (line 208) and in Methods (lines 628-633). As the BLAST process has been updated since then and more sequences have been added over time, we cannot provide a link to the exact search. We have however included accession numbers for all 210 isolates (including those removed due to low quality/duplicates) extracted from the NCBI search in our Supporting Data file 1.

We have added a phylogenetic tree for all of the NCBI sequences where a complete genome was available (Figure S6) as well as a table with ST-group and plasmid typing (Table S8) and a phylogenetic tree of the regions surrounding *tet*(A), including a description of observed regions of direct repeat (Figure S7). The results (lines 231-257), materials and methods (lines 635-657) and supporting results S1 have been modified to include those new analysis. In this process, minor inaccuracies in Fig 2 were identified and corrected. The discussion has also been adapted to present more context regarding the spread of the *tet*(A)$^{\Delta tetR}$ allele (lines 422-424, 472-487 and 520-526). For collection ii, we have clarified in the S1 Results that a phylogeny of all isolates from that collection is available in the original article (as mentioned below). Collection iii is not whole-genome sequenced, only screened by PCR for the presence and allele of *tet*(A), as described on line 210-211, and no phylogeny of these isolates can be presented.

10. S1 results. It'd be helpful to see these isolates on a phylogeny. How many times has the tet(A)ΔtetR allele appeared? Is a wt allele ever regained?

A phylogeny and more in-depth analysis of these isolates can be observed in the referenced original study, which has been clarified in the supplemental text. However, one needs to keep in mind that *tet*(A) is most often carried on transposons and plasmids (as can

now be seen in table S8) and therefore a single event of *tet*(A)$^{\Delta tetR}$ appearance can lead to clonal spread into many different isolates due to HGT. Additionally, none of the collections we used can be considered completely "unbiased", despite our efforts to include as much diversity as we can, and therefore cannot be considered to include all appearences of the *tet*(A)$^{\Delta tetR}$ allele. However, we do agree that a phylogenetic analysis of isolates carrying *tet*(A) alleles, and an analysis of local genetic context of the tet(A) alleles can be beneficial to understanding the spread of the *tet*(A)$^{\Delta tetR}$ allele. We have extended our analysis of our NCBI isolates to consider these suggestions (Figure S6, S7 Table S8). While the number of times the *tet*(A)$^{\Delta tetR}$ has appeared is not exactly clear from these results or our phylogenetic analysis, our analysis indicates a likely small number of occurrences of the mutation in *tetR*(A), which have then spread (see lines 521-526). One hypothesis resulting from our now extended analysis is that the spread of *tet*(A)$^{\Delta tetR}$ might have been facilitated, among other reasons, due to its presence on specific (and possibly highly successufful) plasmids in ST131. Our results and discussion section have been modified to describe our observations and their implications (see previous question for exact lines with modifications).

As described in S1 Results, out of the 38 isolates from the UPEC and blood isolate collection carrying *tet*(A), 14 isolates were identified to carry *tet*(A)$^{\Delta tetR}$. To the best of our knowledge, the *tet*(A)^{wt} allele would only be regained via the renewed uptake of the allele, as reversion of the 24-bp deletion is not to be expected (e.g., instances of deletion of *tet*(A)$^{\Delta tetR}$ or loss of the plasmid carrying *tet*(A)$^{\Delta tetR}$, followed by acquizition of a new plasmid carrying *tet*(A)^{wt}). Analysis of the local genetic context around *tet*(A) (figure S7) seems to show that, indeed, the mutated *tet*(A)$^{\Delta tetR}$ do not seem to spontaneously revert to *tet*(A)^{wt} (which would be a very umprobable genetic event). Loss of *tet*(A)$^{\Delta tetR}$ and acquizition of a new plasmid carrying wild-type *tet*(A) would not be visible in such phylogenetic analysis.

11. Line 231-3. The potential application of this assay seems more suited to the discussion section than the results section.

Comment on potential application has been removed (line 263), as is it is covered in the discussion (lines 543-552), according to the reviewer's suggestion

12. How much time does E. coli spend exposed to tetracycline? The premise is that exposure to low dose tetracyclines selects for the allele with the deletion, but the advantage of the wt allele in the absence of antibiotic suggests that it would have to be rather consistently exposed to antibiotic. Alternatively, it could be that the allele with the deletion may be linked to other factors that confer resistance or fitness advantage or be part of a successful clade. This is another instance where it would be helpful to see the phylogeny to understand how much of the expansion of the allele with the deletion might be due to clonal success vs repeated selection.

This is an interesting question but it is essentially impossible to answer in a quatitative way (as it is for any antibiotic or other selective pressure). What is clear, and as we describe in the discussion section (starting at line 531), is that many environments exist where *E. coli* can be exposed to a tetracycline antibiotic. Apart from exposure in tetracycline-treated humans, where poor drug pharmacokinetics and compliance can lead to low-level antibiotic exposure, another example included in the discussion correspond to past and current use of tetracyclines in agricultural settings that can result in contamination of water and soil. The tetracycline antibiotic doxycycline is also used prophylactically as an antimalarial. While we cannot give an exact description of the length of time that *E. coli* may be exposed to a tetracycline antibiotic, the widespread use and observed environmental contamination strongly suggest

that sub-lethal exposure occurs in many environments. We showed experimentally a fitness advantage of the deletion mutant of several percent (which in an evolutionary context is very high) under such low concentrations of tetracyclines.

Regarding the fitness cost of *tet*(A)$^{\Delta tetR}$ in absence of antibiotic, this cost is very small compared to the fitness advantage at some antibiotic concentrations. While such cost would lead, in the long term, to a decrease in *tet*(A)$^{\Delta tetR}$ frequency (to the benefit of mutants with wild-type *tet*(A)) in environments lacking tetracyclines, this would likely be a very slow process. Furthermore, reversion of *tet*(A)$^{\Delta tetR}$ to *tet*(A)^{wt} is unlikely due to the nature of the mutation (deletion), and acquizition of a new plasmid carrying wild type *tet*(A), which is more likely, comes with its own associated cost and would result in different selection dynamics. Finally, our now extended analyses of phylogeny and local genetic contexts around *tet*(A) also point to clonal spread of the mutated *tet*(A)$^{\Delta tetR}$, as well as its association with some specific plasmids which might be particularly successful in ST131. Thus, it is clear that additional selection forces are also involved in the spread of the mutated allele, and the observed frequent occurrence of *tet*(A)$^{\Delta tetR}$ among ST131 indicates that those selection forces likely affect the observed small cost of *tet*(A)$^{\Delta tetR}$ in absence of tetracyclines. With this updated information, we have contextualized our statements regarding the dynamics of selection of the allele in the discussion (lines 472-487, 517-530), as well as changed our title and some statements in the abstract (lines 34-36, 38-39) and hope this sufficiently addresses your concerns.

13. Line 333. I think this should cite Fig S5, not S6.

This error has been corrected.

14. The regular appearance of the 24bp deletion in tetR(A) even in the absence of antibiotic pressure calls into question the interpretation that it's only selected for under low antibiotic pressure (lines ~478-482).

This is also an interesting point that is quite difficult to address experimentally. One possibility for the regular occurrence of the deletion also in absence of drug could be that the 24-bp deletion in *tetR*(A) occurs at a high rate (discussed earlier,lines 497-501, 514-517). However, our analysis of local genetic contexts around *tet*(A) (Figure S7) points towards a low frequency of spontaneous deletions in *tetR*(A) and the spread of a limited amount of mutated alleles. This along with our observation of occasional *tet*(A)$^{\Delta tetR}$ selection in absence of tetracyclines in our evolution experiments, which is unexpected and contradictory to our competition experiments, indicates that selection dynamics in our evolution experiments are more complex than expected from our competition experiments, and that results of the evolution experiment do not all seem to match with what we can observe when analysing isolates. We think that this is a good example of how difficult it can be to interprete laboratory results and extrapolate them to the reality of nature. We have further emphasized the differences observed between experiments and observation in the discussion (lines 517-530).

15. Lines 345 and 365. Was it 192 or 196 generations?
This error has been corrected to 196 generations (line 397).

16. Lines 394-401. What evidence is there that the four isolates described here lost the amplifications as opposed to some other process—e.g., something technical like testing or recording error vs some other genetic alteration? Had the authors observed

evidence of loss of amplifications in the strains they were working with, or do the authors have other evidence to suggest the amplifications are unstable?

While we do not have any specific evidence of amplifications that have been lost in this case, it has been shown previously in several studies that such amplifications are unstable and can segregate rapidly. We have added an additional reference to previous work elucidating the unstable nature of such amplifications and clarified in the text that amplifications were not observed in these strains, only hypothesized (lines 433-436).

17. Lines 402-4. I'm confused by claim that this is the first instance of considering genetic diversity in evolution of antibiotic resistance. See, for example, https://www.pnas.org/doi/10.1073/pnas.2016886118, and the citations therein for just some instances of preceding work demonstrating this point, at least as far as I understand the authors. In what way is this first?

While work such as the article cited by the reviewer is an example of increased genetic diversity in studies of antibiotic resistance, the suggested work and the citations included within it largely look at descendants of a single isolate, evolved in identical or similar environments. Our work analyzes isolates from diverse sources, including geographically, that have been exposed to different environments and pressures, and most likely better represent the genetic diversity as it can be found in nature for a bacterial species with an open pan-genome such as *E. coli.* We have clarified this point (line 439).

18. Lines 424-7. Please show this as a supplementary figure and put it in the results section. An assessment of the structural impact of the mutation seems appropriate for the first description of the mutation.

Collaborators are currently performing X-ray crystallography structural analysis of the mutated TetR(A) with/without tetracyclines bound, and this work will provide a detailed analysis of the impact of the deletion mutation. However, we have added a figure (S10) showing the protein structure of wild-type TetR(A) bound to tetracycline with the 8 amino acids deleted in the mutated TetR(A) highlighted, as well as the dimerization site and the C9 site on tetracycline. This is referenced in the discussion (line 463) however, as we have not yet done any structural analysis of our own and can only hypothesize on the effect of the deletion in the discussion.

19. Line 433. The question, "why is this deletion prevalent in clinical isolates" raises the issue of what should be considered 'prevalent' and the issues about assessing collections as in my comment above.

“Prevalence” has been changed to occurrence in accordance with comments from another reviewer, and we believe the frequencies of occurrence of the allele that we observe are high. We hope our previous clarifications regarding the sequences from NCBI, the UPEC and blood isolate collection from Salipante et al, the association with ST131 and specific plasmids, and our modifications to the results (Lines 231-247) and contextualization of the discussion (lines 472-487, 517-530) acknowledge these concerns.

20. Section starting line 484. The screen to predict TGC resistance through this path is a bit confusing. Is the idea that an E. coli reported as susceptible to TGC that also has

wildtype tet(A) is likely to develop resistance if standard dosing is used? How different is this rate from what's observed in E. coli lacking tet(A)?

We have clarified the reasoning behind the PCR screen and also that further experiments are necessary to confirm the clinical usefulness of our PCR screen (Lines 543-552). We have shown in this work that isolates that are initially susceptible to TGC but carry wild-type *tet*(A)^{wt} have a high frequency of TGC-resistant mutants *in vitro* that is several log higher than that of isolates without *tet*(A) or with *tet*(A)$^{\Delta tetR}$ (Table S2). Furthermore, we also show in a subset of isolates carrying *tet*(A)^{wt} that they can further evolve MIC levels well above clinical resistance levels, reaching MICs well above what can be reached using, for example, isolate MG1655.

Reviewer #3:

1. A revision of the subsections and figures would help to follow the paper. Different sections of the paper refer to the same figures that do not always follow an order. This would have to be carefully revised in order to facilitate the reading.

We have worked to improve the organization and structure of the figures to simplify for the reader. Figures S2, S3, and S4, and S5 in the original submission have been rearranged and reordered to better fit the flow of the text. Please see the last comment for more specific requested changes regarding the order and structure of tables and figures.

2. Lines 159-166 and Figure S2C. About Klebsiella pneumoniae isolates resistant to tigecycline and omadacycline. Isolates DA32094 and its spontaneous TGC-resistant mutant DA59363 are only mentioned in Figure S2C. The names of the strains could be also included in table S1 and in the text in order to better follow the text. Please, explain or support by appropriate reference how this "spontaneous mutation" occurs. This is a very important result that is barely exploited.

We have edited the text in this section (lines 168-170) to clarify that the omadacycline testing was performed on *E. coli*, not *K. pneumoniae*. Table S1 is describing the phylogenetic diversity of the original *E. coli* isolates screened and not all isolates. All of the strains and mutants are described in Table S9 in the original submission, which was maybe not available to the reviewers as it was a separate excel files and other separate files were uploaded to PLOS but do not seem to have been provided to the reviewers. We apologize for this confusion.

There are numerous publications and reviews with proposed mechanisms for tandem genetic amplifications. The proposed mechanisms involve large repeated sequences present in the same orientation and that end up delimiting the amplified repeat (see for example Fig. 2). In our manuscript, we have now searched for such repeats around *tet*(A) in numerous isolates (see S7 Fig., lines 248-257 and 422-424) to highlight the potential for *tet*(A)^{wt} amplification in numerous isolates, and we refer to a review about genetic amplifications where the readers can learn more about mechanisms of amplification (reference #32).

3. Lines 179-184. The tandem amplification of the tet(A) using other antibiotics such as tobramycin is really interesting and is not exploited by the authors either. Note that the MDR island of IncFII derivatives of R1 comprises Tn1721 and also other

transposons encoding antibiotic resistance. Did you try other antibiotics such as beta-lactams or chloramphenicol or quinolones? Selection by antibiotics that act on different cell targets would contribute to understanding the evolution and prediction of tigecycline resistance. Some of the antibiotics mentioned are the first election drugs to treat infections caused by enterobacterales.

This isolate (DA33135) and its derivative mutants are from a different study, as described in the text (lines 189-191). TGC resistance was not the focus of the original article. As described in the text, in the mutants of DA33135, amplifications were selected in presence of tobramycin due to amplification of an aminoglycoside resistance gene. The amplified unit in those mutants also happen to carry *tet*(A)$^{\Delta tetR}$, which got co-amplified. This isolate and its derivative mutants are simply used in our study as a way to identify if increased gene dosage through amplification would lead to increased TGC resistance in an isolate carrying *tet*(A)$^{\Delta tetR}$ when we could not select mutants with amplifications of *tet*(A)$^{\Delta tetR}$ when selection was performed with TGC.
We agree that the occurrence of amplifications carrying multiple resistance genes conferring resistance to multiple classes of antibiotics is interesting as it could lead to co-amplification of *tet*(A)^{wt} - and therefore co-selection for tigecycline resistance - during treatment with other drugs. This could be studied further but we think it is outside of the scope of this study.

4. Lines 221-225 (results) and lines 439-449 (discussion). The idea of linking the success of this mechanism of tigecycline resistance to the success of widespread clones such as ST131 is tempting. However, the occurrence of these mutants might be linked to the widespread of the IncFII plasmid where Tn1721-tet(A)is located. This plasmid IncFIIpC15-1a-like and many similar derivatives are overrepresented in ST131. An MDR island located in this type of FII plasmids is a more (or equally) plausible explanation.

In accordance with comments from another reviewer, we have performed a more extensive analysis of the NCBI isolate collection and included plasmid typing, comparison of local genetic context around *tet*(A) and identification of direct repeats surrounding *tet*(A) to address reviewers concerns (lines 231-257 , S1 results, Figure S6 and S7, and Table S8). The analysis further confirms clonal pread as well as, as speculated by reviewer 3, linkage of *tet*(A)^{wt} and *tet*(A)$^{\Delta tetR}$ to specific but different IncF plasmids (according to plasmid typing) in ST131 clones. We refer to previous work regarding successful plasmids in ST131 and hypothesize that the plasmids carrying *tet*(A)$^{\Delta tetR}$ might be particularly successful in ST131, and therefore that the possible association of *tet*(A)$^{\Delta tetR}$ with successful plasmids might play a role in the unusual high frequency of that allele in ST131 *vs* other ST clones. We have stated more clearly this in the discussion (lines 478-485) and generally conextualized our text regarding these findings.

5. Lines 227-233. This small paragraph may be a conclusion /recommendation you can extract from your results. I would strongly recommend only maintaining this point in the discussion section

We have kept only our findings regarding the accuracy of the screen in the results (line 263) and removed the comment on potential application, as is it is covered in the discussion of the screen (lines 543-555).

6. Lines 461. The hypothesis of clonal interference when plasmids are involved is

difficult to maintain. Note that E. coli populations with and without plasmids often coexist in human and environmental samples and also coexist with other enterobacterales that may carry or acquire the same plasmids. Other phenomena such as bet-hedging or frequent dependent selection, more associated with the maintenance of the robustness of the populations, could also explain this

These experiments were performed with *tet*(A) cloned on the chromosome and thus do not involve plasmids. Therefore, horizontal transfer of alleles between populations is not happening and interfering with our data. In this section, we only try to describe that other beneficial mutations (e.g. media adaptation) than the 24-bp deletion in *tetR*(A) might appear during our evolution experiment and as a result generate a complicated dynamics. In this type of evolution experiment, when one starts with a wild type population there is a "waiting time" for the first spontaneous deletion mutant to occur (this is stochastic and will follow a Luria-Delbruck dynamics). When this mutation has occurred the take-over time will be determined by the difference in growth rate (selection coefficient, which is about 5% in this experiment) between the deletion allele and other populations present in the medium. However, because the vast majority of bacterial cells carry *tet*(A)^{wt} for most of the length of the evolution experiment, the unrelated beneficial mutations will likely occur in bacteria carrying the wild-type *tet*(A) allele. Such mutations will therefore change the competition dynamics by decreasing the growth advantage that the deletion in *tetR*(A) confers, slowing down the ability of isolates with deletions in *tetR*(A) from outcompeting those fitter mutants. Those fitter mutants do not affect the competition experiments where isolates with *tet*(A)^{wt} and *tet*(A)$^{\Delta tetR}$ are initially present at equal proportions, and in otherwise isogenic strains. We have clarified that part in the discussion (lines 509-514).

7. Line 246. Please, explain "possibly" here. This result should be "black/white". If not, it should be discussed.

We have edited the text to clarify the results described here (lines 276-278) and remove any ambiguity.

8. Change "prevalence" by "occurrence". You do not can distinguish the cases by date-onset and the concepts of "prevalence" and "incidence" relies on the temporal parameter.

We have changed "prevalence" to either "occurrence", "frequency", "common" or some variation thereof, depending on the context, throughout the text.

9. Tables and figures. This part needs careful revision. Below, I raise the more relevant changes

- **Tables S2 and S4 are missing. At least, this reviewer could not find any excel file. If they are there, forgot the comment.**

Tables S2 and S4 were intended to be included as separate Excel files, as they become very difficult to manage as PDF files. We apologize to the reviewer that these tables were not provided to them.

- **Table S3. This table is difficult to read. If the issue is to show the phenotype diversity of the two main genotypes, the main criterium is the genotype and this**

must be the first column. Then, this column should show the two genotypes, first all tet(A) and afterward, all tet(A) deleted. This reorganization would help to process the information.

Table S3 has been adapted in accordance to the reviewer's comments.

- **Figure 3. This figure is very confusing and should be revised. Panel D cannot be an individual panel; it must be the footnote of the figure because explains panels A-C.**

Figure 3 has been adapted. Panels A-D have been combined into one panel, with a clearer description of the groups in the figure to simplify for the reader.

Academic editor:

We have included this article in our discussion (lines 566-568)

General comments:

Supplemental numerical data was added (Supplemental Data File 1)
