# Supplementary material for: Low levels of tetracyclines select for a mutation that prevents the evolution of high-level resistance to tigecycline
Source: PLoS Biol. 2022 Sep 28;20(9):e3001808. doi: 10.1371/journal.pbio.3001808 (PMC9550176; doi:10.1371/journal.pbio.3001808)
Supplement: Attachment — Submitted filename: Response_to_reviewers_second_revision.pdf [file pbio.3001808.s031.pdf]

Low levels of tetracyclines select for a mutation that prevents the evolution of high-level resistance to tigecycline

J. Jagdmann, D.I. Andersson and H. Nicoloff.

Answers to reviewers and Editor's remarks.

General points:

a) Please address the remaining points from reviewer #2, and the comments from the Academic Editor that appear at the foot of this email.

We addressed each point, and describe them specifically below.

b) Given the comments from the reviewer and the Academic Editor, plus the need to make both the specific and conceptual advance more explicit in the Title, we suggest that you change the Title to something like "Low tigecycline levels select for a mutation that prevents the evolution of high-level resistance to this antibiotic" (but feel free to suggest your own version).

We have suggested a more specific title, while still including the concept of several tetracycline antibiotics selecting for the mutation at low levels.

c) Many thanks for providing the underlying data. Please could you also cite its location clearly in each relevant main and supplementary Figure legend, e.g. "The data underlying this Figure may be found in S1 Data."

We have added a reference to each relevant figure legends (see lines 129, 225-226, 303, 360-361 as well as in legends of Tables S2, S3, S7, S8 and Figures S2, S3, S4, S9 and S11).

Reviewer #2:

I thank the authors for their responses and work to update the manuscript. My main comment is that the title and abstract are a bit too general. The mutation they've identified is not only spread clonally, so describing this as 'clonal spread' seems misleading. Similarly, "a mutation preventing the evolution of high-level resistance" seems incomplete -- I suggest adding that this is high-level resistance specifically of tigecycline and in E. coli. In the abstract, the added language, 'we found evidence of clonal spread...' should also include mention that not all the spread was clonal, as noted in the discussion: "However, we also observed that isolates carrying tet(A)ΔtetR belonged not only to ST131 but also several other ST groups". Also in the abstract, I wonder about the addition of 'clonal spread' on line 38. As it reads, it suggests that clonal spread selected for a mutation, whereas what I think the authors mean is that clonal spread increased the frequency of representation in the databases they checked. I suggest revising this sentence.

We have made the title more specific, but did not add *E. coli* in it as the amplifications could also be selected in other organisms such as *K. pneumoniae*. We have modified the abstract on lines 35-36 and 39 to clarify the impact of the observed clonal spread according to the reviewer's recommendations.

Academic Editor:

a) I checked the authors' response also to rev#3 and think this is satisfying. I agree with the authors that their request for further experiments with tobramycin is beyond the scope of the current work.

b) I further agree with rev#2 that "clonal spread" should better be removed from the title and the role of clonal spread could be clarified in the abstract (although it will be clear to the reader that the data presented suggests at least a positive role of clonal spread of ST131).

We have altered the title according to suggestions and edited the abstract (lines 35-36 and 39) to better clarify the role of ST131 clonal spread.

c) It would help to make explicit in Fig. 1B that the presented mutant frequencies refer to a fluctuation assay, not to frequencies among (clinical) isolates

This clarification has been made in the figure text for 1B Fig (line 123).

d) I spotted several typo's (e.g., a reference to Fig.2E -- which doesn't exist -- in line 376.

We have attempted to find and correct typos thoughouut the text.

Changes to the reference list:

Reference number 63 has been edited.
